# Seroprevalence of severe acute respiratory coronavirus virus 2 (SARS-CoV-2) antibodies among healthcare personnel in the Midwestern United States, September 2020–April 2021

**DOI:** 10.1017/ash.2022.375

**Published:** 2023-08-04

**Authors:** Rachel E. Bosserman, Christopher W. Farnsworth, Caroline A. O’Neil, Candice Cass, Daniel Park, Claire Ballman, Meghan A. Wallace, Emily Struttmann, Henry Stewart, Olivia Arter, Kate Peacock, Victoria J. Fraser, Philip J. Budge, Margaret A. Olsen, Carey-Ann D. Burnham, Hilary M. Babcock, Jennie H. Kwon, for the CDC Prevention Epicenters

**Affiliations:** 1 Division of Infectious Diseases, Department of Medicine, Washington University School of Medicine, St. Louis, Missouri; 2 Department of Pathology and Immunology, Washington University School of Medicine, St. Louis, Missouri

## Abstract

**Objective:**

To determine the prevalence of severe acute respiratory coronavirus virus 2 (SARS-CoV-2) IgG nucleocapsid (N) antibodies among healthcare personnel (HCP) with no prior history of COVID-19 and to identify factors associated with seropositivity.

**Design:**

Prospective cohort study.

**Setting:**

An academic, tertiary-care hospital in St. Louis, Missouri.

**Participants:**

The study included 400 HCP aged ≥18 years who potentially worked with coronavirus disease 2019 (COVID-19) patients and had no known history of COVID-19; 309 of these HCP also completed a follow-up visit 70–160 days after enrollment. Enrollment visits took place between September and December 2020. Follow-up visits took place between December 2020 and April 2021.

**Methods:**

At each study visit, participants underwent SARS-CoV-2 IgG N-antibody testing using the Abbott SARS-CoV-2 IgG assay and completed a survey providing information about demographics, job characteristics, comorbidities, symptoms, and potential SARS-CoV-2 exposures.

**Results:**

Participants were predominately women (64%) and white (79%), with median age of 34.5 years (interquartile range [IQR], 30–45). Among the 400 HCP, 18 (4.5%) were seropositive for IgG N-antibodies at enrollment. Also, 34 (11.0%) of 309 were seropositive at follow-up. HCP who reported having a household contact with COVID-19 had greater likelihood of seropositivity at both enrollment and at follow-up.

**Conclusions:**

In this cohort of HCP during the first wave of the COVID-19 pandemic, ∼1 in 20 had serological evidence of prior, undocumented SARS-CoV-2 infection at enrollment. Having a household contact with COVID-19 was associated with seropositivity.

Severe acute respiratory syndrome coronavirus 2 (SARS-CoV-2), the causative agent of coronavirus disease 2019 (COVID-19), has resulted in substantial morbidity and mortality since its emergence.^1^ An estimated 35.1% of SARS-CoV-2 infections are asymptomatic,^2^ yet even asymptomatic infections are not benign. Mathematical modeling suggests that nearly 25% of all SARS-CoV-2 transmission has been attributed to people with asymptomatic infections.^3^ In healthcare settings, these asymptomatic infections may put vulnerable patients and critical staff at risk. Furthermore, long-term sequelae can occur following asymptomatic and mild infections.^4^ Thus, mild or asymptomatic COVID-19 may have consequences for both individuals and communities.

The seroprevalence of antibodies to the SARS-CoV-2 nucleocapsid (N) protein provides insight into the proportion of people who have experienced SARS-CoV-2 infection.^5,6^ Healthcare personnel (HCP) who work in COVID-19 units or who care for known or suspected COVID-19 patients may be considered high-risk for exposure to SARS-CoV-2, although appropriate use of personal protective equipment mitigates that risk.^7^ The objective of this study was to determine the prevalence of SARS-CoV-2 IgG N-antibodies among high-risk HCP with no known history of COVID-19 and to identify potential risk factors of seropositivity.

## Methods

### Study design and participants

This prospective cohort study was conducted at a large academic medical center in St. Louis, Missouri. Participants were HCP aged ≥18 years, who were employed at Barnes-Jewish Hospital, St. Louis Children’s Hospital, or Washington University School of Medicine in St. Louis. HCP were eligible to participate in the study if they provided cared for COVID-19 patients. HCP with nondirect patient-care roles who handled specimens with potential SARS-CoV-2 (eg, laboratory personnel) or who worked in a COVID-19 ward or ICU (eg, dining services, pharmacist, dietitian) were also eligible to participate. HCP who were participating in a COVID-19 vaccine trial and those with a history of COVID-19, diagnosed via a positive SARS-CoV-2 PCR, antigen, or serologic antibody test, were excluded.

Participants were recruited via posters placed in staff areas on the medical center campus and recruitment visits to various wards and departments where staff would be expected to have cared for COVID-10 patients. No monetary incentive was offered to participants; however, participants were informed of their study serologic testing results.

The study protocol was reviewed and approved by the Washington University Human Research Protection Office. All participants provided written informed consent. Participants completed 2 study visits (enrollment and follow-up). At each visit, a blood specimen was obtained and HCP completed a survey to provide information about demographics, job characteristics, pre-existing medical conditions, known and potential SARS-CoV-2 exposures, use of social distancing and face masks at work and outside work, and COVID-19–compatible symptoms. At the follow-up visit, HCP were also asked about any SARS-CoV-2 testing that occurred since the enrollment visit and about SARS-CoV-2 vaccination. Not all participants who had blood drawn at the follow-up visit completed the follow-up survey. Enrollment visits were conducted between September 22, 2020, and December 1, 2020. Follow-up visits were scheduled 70–160 days after the enrollment visit and took place between December 8, 2020, and April 27, 2021.

### Antibody testing

Blood specimens were collected in a 10-mL K_2_ EDTA tube and were stored at room temperature for up to 8 hours before refrigeration. Specimens were centrifuged, and plasma aliquots were stored for up to 4 days at 4°C prior to analysis. IgG anti–N-antibodies were detected in plasma samples using the Abbott SARS-CoV-2 IgG assay on the Abbott Architect i2000 (Abbott Laboratories, Abbott Park, IL), according to the manufacturer’s instructions. Results were calibrated with a relative light unit calibrator, which was used to calculate a ratio of specimen absorbance to calibrator absorbance. Samples with an index specimen/calibrator (S/C) value ≥1.4 were interpreted as reactive (seropositive).

### Statistical analyses

We used χ^2^ and Fisher exact tests to examine associations between N-antibody test results (positive vs negative) and HCP characteristics, SARS-CoV-2 exposure history, and symptoms. Odds ratios (ORs) and 95% confidence intervals (CIs) were estimated using univariate logistic regression. Wilcoxon rank-sum tests and Wilcoxon signed-rank tests were used to evaluate differences in median N-antibody signal at enrollment and follow-up. Statistical analyses were performed using SAS version 9.4 software (SAS Institute, Cary, NC), and *P* < .05 was considered statistically significant.

## Results

### Cohort characteristics

In total, 400 HCP who worked in a clinical setting with COVID-19 patients or specimens had enrollment blood specimens collected for quantification of SARS-CoV-2 IgG N-antibodies. The study cohort was predominately female (63.8%) and white (79%), with a median age of 34.5 years (IQR, 30–45) (Table 1). The cohort consisted primarily of physicians (44.8%) and other HCP (44.0%) in direct patient-care roles (eg, nurses, physician assistants, respiratory therapists) (Table 1). Also, 44 HCP (11.0%) reported indirect patient-care roles, which included administration, dieticians, dining services personnel, laboratory personnel, pharmacists, and speech therapists.

**Table 1.** Participant Survey Responses at Enrollment

Characteristic	Frequency (N = 400), No. (%)
**Demographics**	
Age, median [IQR]	34.5 [30–45]
Sex, female	255 (63.8)
**Race^a^**	
Asian	50 (12.5)
Black	26 (6.5)
White	316 (79.0)
Other	13 (3.2)
Hispanic ethnicity	12 (3.0)
**Comorbidities**	
Seasonal allergies	178 (44.5)
Obesity	52 (13.0)
Cerebrovascular disease	23 (5.7)
Use of immunosuppressive medications	15 (3.7)
Pregnancy	13 (3.2)
Lung disease	11 (2.7)
**Employment information**	
**Primary work assignment**	
Intensive care unit	39 (9.7)
Medical/surgical wards/specialties	200 (50.0)
Pediatrics	44 (11.0)
Emergency services	73 (18.3)
Pathology/pharmacy/radiology/laboratory	33 (8.3)
Hospital staff services^b^	5 (1.3)
Not applicable/missing	6 (1.5)
**Job role**	
Physician^c^	179 (44.8)
Other direct patient care role^d^	176 (44.0)
Indirect patient-care role^e^	44 (11.0)
Missing	1 (0.3)
**Commute method**	
Drive a car alone	346 (86.5)
Walk or bike	44 (11.0)
Other transportation method^f^	10 (2.5)

a Participants could select multiple responses for this question.

b Hospital staff services include: food and nutrition services and mechanical assistant.

c Physicians included: attendings (n = 98), residents (n = 46), and fellows (n = 35).

d Other direct patient care roles included nurse (n = 88), advance practice nurse/nurse practitioner/physician assistant (n = 53), nurse assistant/patient care technician (n = 10), respiratory therapist (n = 10), medical student (n = 7), paramedic (n = 4), and physical or occupational therapist (n = 4).

e Indirect patient-care roles included clinical laboratory personnel (n = 26), administration (n = 5), pharmacist (n = 3), dietician (n = 2), dining services personnel (n = 2), clinical research staff (n = 2), EMT student (n = 1), child life specialist (n = 1), CT technologist (n = 1), and speech therapist (n = 1).

f Other transportation methods includes bus, carpool, and train.

### Seropositivity at enrollment

At the time of study enrollment, 18 participants (4.5%) were seropositive despite having no reported history of a positive SARS-CoV-2 test. Median IgG N-antibody levels were 3.06 S/C for seropositive HCP versus 0.03 S/C for seronegative HCP. Seropositive and seronegative HCP did not differ in age [median, 34 years (IQR, 30–35) vs 39.5 years (IQR, 28–50); Wilcoxon rank-sum *P* = .70,] or other demographic factors (Table 2). Seropositivity was also not associated with having a direct versus indirect patient-care role (OR, 0.62; 95% CI, 0.17–2.22) or with greater frequency of contact with COVID-19 patients (OR, 2.43; 95% CI, 0.94–6.31) (Table 2).

**Table 2.** Risk Factors for a Seropositive Antibody Test Result at Enrollment in Bivariate Analysis (N = 400)

Characteristic	Seropositive (n = 18)	Seronegative (n = 382)	OR (95% CI)	*P* Value
**Demographics**				
Age, median [IQR]	39.5 [28–50]	34.0 [30–45]	Not applicable	.69
Sex, female	13 (72.2)	242 (63.4)	1.50 (0.53–4.31)	.45
Race, nonwhite	3 (16.7)	86 (22.5)	0.69 (0.19–2.43)	.21
Direct patient-care role	15 (83.3)	340 (89.0)	0.62 (0.17–2.22)	.20
**Occupational risk factors and precautions**				
Known exposure to coworker with COVID-19 in the past 30 d^a^	5 (27.8)	44 (11.5)	2.94 (1.00–8.63)	.04
Contact with known or suspected COVID-19 patients >50% of the time while at work^a^	10 (55.6)	129 (33.8)	2.43 (0.94–6.31)	.06
Unable to practice social distancing when at work and not involved in immediate patient care >50% of the time	7 (38.9)	197 (51.6)	0.60 (0.23–1.57)	.29
Wears a face mask ≤50% of the time while at work^b^	1 (5.6)	5 (1.3)	4.44 (0.49–40.08)	.22
**Nonoccupational risk factors**				
Household member with known or suspected COVID-19 in the past 30 d^c^	4 (22.2)	8 (2.1)	13.32 (3.58–49.54)	.001
Other known, specific COVID-19 exposure outside work (excluding sick household member) in the past 30 d^c^	0 (0.0)	9 (2.4)	Undefined	1.00
Practices social distancing when in public ≤50% of the time^c^	0 (0.0)	34 (8.9)	Undefined	1.00
Wears a face mask ≤50% of the time while in public^c^	0 (0.0)	19 (5.0)	Undefined	1.00
**Activities in the past 30 d**				
Travel^d^	1 (5.6)	92 (24.1)	0.18 (0.02–1.40)	.09
Attended a large gathering	8 (44.4)	172 (45.0)	0.98 (0.38–2.53)	.96
Ate indoors at a restaurant	8 (44.4)	109 (28.5)	2.00 (0.77–5.21)	.15
Ate outdoors at a restaurant	8 (44.4)	170 (44.5)	1.00 (0.39–2.58)	1.00
Visited a medical office	8 (44.4)	159 (41.6)	1.12 (0.43–2.91)	.81
Visited a store	17 (94.4)	367 (96.1)	0.70 (0.09–5.57)	.37
**Symptoms**				
Had illness thought to potentially be COVID-19 since February 2020^d^	10 (55.6)	59 (15.4)	6.82 (2.59–18.00)	<.001

Note. IQR, interquartile range; PPE, personal protective equipment; ICU, intensive care unit.

a Two HCP in the seronegative group were missing a response to this question.

b During the study period, PPE recommendations varied by job and location. Cloth masks were recommended for nonclinical staff, surgical masks were recommended in most clinical spaces, and N95 masks were recommended in COVID-19 ICUs and during aerosol-generating procedures. During the study period, our facility faced PPE shortages that were consistent with nationwide PPE shortages.

c One HCP in the seronegative group was missing a response to this question.

d Three HCP in the seronegative group were missing a response to this question.

Having a household member with known or suspected COVID-19 in the past 30 days was strongly associated with seropositivity at enrollment (OR, 13.32; 95% CI, 3.58–49.54). Other positive associations were exposure to a coworker with COVID-19 within the past 30 days (OR, 2.94; 95% CI, 1.00–8.63) and reporting an illness thought to have possibly been COVID-19 at any time since February 2020, without having had a positive SARS-CoV-2 test (OR, 6.82; 95% CI, 2.59–18.00) (Table 2). There were no significant associations between antibody test result and use of social distancing or a mask while at work, recent travel, or having attended a large gathering, eaten at restaurants, or visited stores or medical offices in the previous 30 days (Table 2). Moreover, 91 HCP (22.8%) reported COVID-19–compatible symptoms (eg, fever, chills, cough, shortness of breath, etc) occurring in the 14 days prior to the enrollment visit, 5 of whom were seropositive.

### Seropositivity at follow-up

Of the 400 HCP who completed a study enrollment visit, 309 (77.3%) returned for a follow-up visit. The median time between enrollment and follow-up visits was 77 days (IQR, 70–86; range, 67–153). Among those HCP who completed a study follow-up visit, 34 (11.0%) were seropositive, 9 of whom (26.5%) had also been seropositive at their enrollment visit (Supplementary Fig. 1).

**Figure 1.**
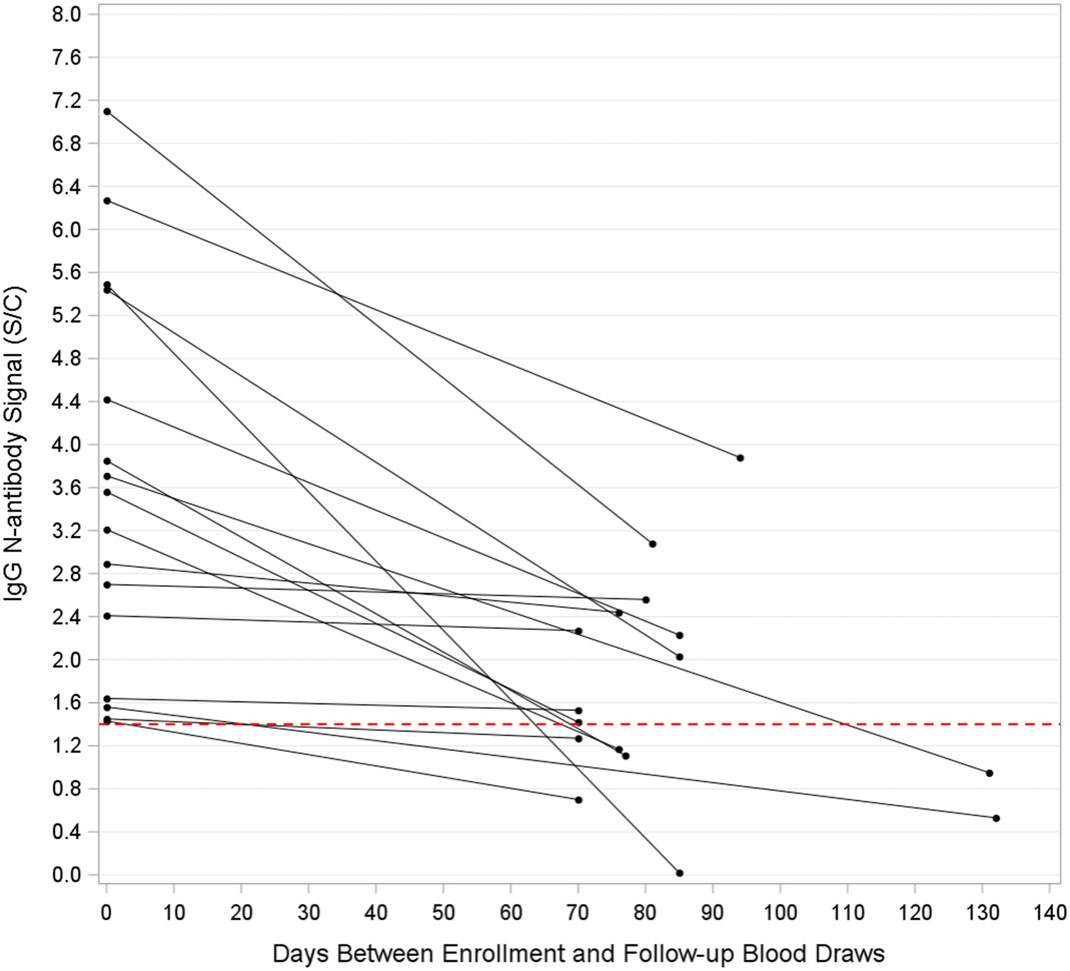
Change in IgG N-antibody signals at enrollment versus follow-up among HCP who were seropositive at enrollment and who completed a follow-up visit (n = 16). The dotted line represents the seropositivity threshold (index specimen/calibrator value ≥ 1.4). In total, 7 HCP experienced seroreversion at follow-up, and 9 remained seropositive.

Of the 18 HCP who were seropositive at enrollment, 16 completed a follow-up visit. Among them, 7 (43.8%) were seronegative at follow-up, while 9 (56.3%) remained seropositive (Fig. 1). Median IgG anti-N antibody signals among persistently seropositive HCP declined from 3.38 S/C at enrollment to a median of 1.47 S/C at follow-up (Wilcoxon signed-rank test, *P* = .001). All 9 seropositive HCP showed a decrease in antibody signal between enrollment and follow-up (Fig. 1).

**Figure 2.**
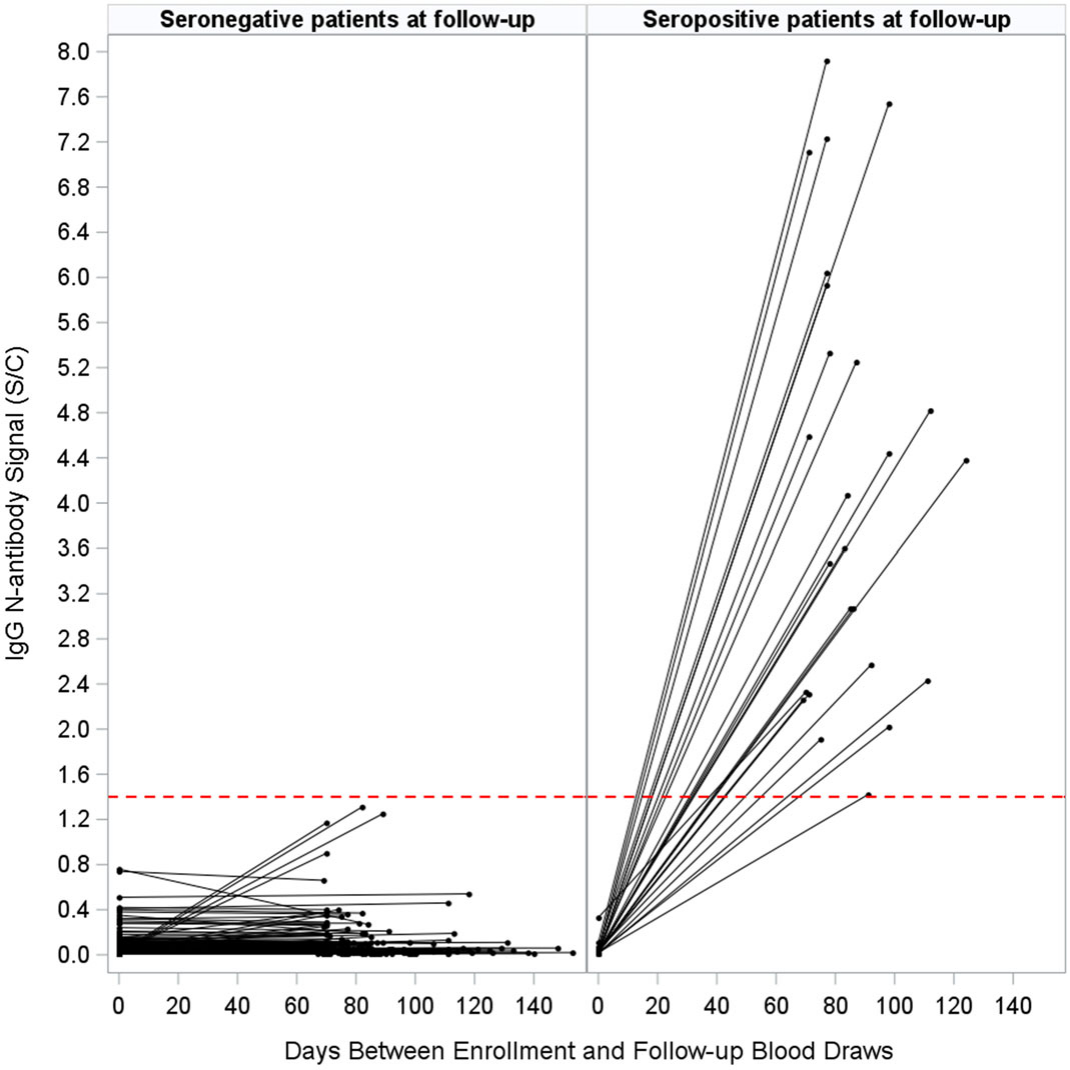
Change in IgG N-antibody signals at enrollment versus follow-up among participants who were seronegative at enrollment and who completed a follow-up visit (n = 293). The dotted line represents the seropositivity threshold (index specimen/calibrator value ≥ 1.4). Overall, 25 HCP who were seronegative at enrollment and had seroconverted at follow-up (panel A), and 268 remained seronegative (panel B).

In total, 293 HCP who were seronegative at enrollment completed a follow-up visit. Between enrollment and follow-up, 25 (8.5%) had converted from seronegative to seropositive while 268 (91.5%) remained seronegative (Fig. 2). Of the 25 participants that seroconverted (seronegative to seropositive), 22 completed a follow-up survey; of these 16 (72.7%) reported having had a positive SARS-CoV-2 PCR test between enrollment and follow-up. In addition, in the time between enrollment and follow-up, 13 HCP who converted from seronegative to seropositive (59.1%) reported a household COVID-19 exposure, 7 (31.8%) reported a specific COVID-19 exposure while at work, and 6 (27.3%) reported a specific COVID-19 exposure outside of work (excluding infected household members).

Of the 309 HCP who returned for a follow-up study visit, 13 participated in antibody testing but did not complete a follow-up survey and were therefore excluded from the risk-factor analysis; 3 were seropositive at follow-up and 10 were seronegative at follow-up. Among the 296 HCP included in the follow-up visit risk factor analysis, 31 (10.5%) were seropositive, 9 of whom had also been seropositive at baseline (29.0%). Also, 40 HCP (13.5% of those with follow-up serology and survey data) reported having had an illness thought to be COVID-19 between enrollment and follow-up. These HCP had increased risk of being seropositive (OR, 5.64; 95% CI, 2.46–12.93) compared to HCP reporting no such illness (Table 3). Having a positive PCR test between enrollment and follow-up was also associated with being seropositive at follow-up (OR, 46.04; 95% CI, 15.75–134.64) (Table 3).

**Table 3.** Risk Factors for a Seropositive Antibody Test Result at Follow-up (70–160 Days After Enrollment Visit) in Bivariate Analysis (N = 296)

Risk Factor	Seropositive (n = 31)	Seronegative (n = 265)	OR (95% CI)	*P* Value
**Occupational risk factors**				
Known, specific COVID-19 exposure while at work^a^	9 (29.0)	60 (22.6)	1.46 (0.64–3.36)	.37
Contact with known or suspected COVID-19 patients >50% of the time while at work^b^	15 (48.4)	95 (35.8)	1.78 (0.83–3.80)	.14
Unable to practice social distancing when at work and not involved in immediate patient care >50% of the time	14 (45.2)	116 (43.8)	1.06 (0.50–2.24)	.88
Wears a face mask ≤50% of the time while at work	0 (0.0)	6 (2.3)	undefined	1.00
**Nonoccupational risk factors**				
Household member with known or suspected COVID-19 since the enrollment visit^c^	13 (41.9)	19 (7.2)	9.35 (3.99–21.93)	<.001
Other known, specific COVID-19 exposure outside of work (excluding sick household member) since the enrollment visit^c^	6 (19.4)	14 (5.3)	4.31 (1.52–12.19)	.009
Travel in the past 30 d	8 (25.8)	62 (23.4)	1.14 (0.49–2.67)	.77
Attended a large gathering in the past 30 d	6 (19.4)	82 (30.9)	0.54 (0.21–1.36)	.19
Ate indoors at a restaurant in the past 30 d	8 (25.8)	61 (23.0)	1.16 (0.50–2.73)	.73
Ate outdoors at a restaurant in past 30 d	4 (12.9)	58 (21.9)	0.53 (0.18–1.57)	.25
Visited a medical office in past 30 d	8 (25.8)	117 (44.2)	0.44 (0.19–1.02)	.06
Visited a store in past 30 d	29 (93.5)	252 (95.1)	0.75 (0.16–3.48)	.66
Visited other public location in past 30 d	17 (54.8)	137 (51.7)	1.14 (0.54–2.40)	.74
**COVID-19 symptoms and testing**				
Had illness thought to be COVID-19 since enrollment visit^a,c^	12 (38.7)	28 (10.6)	5.64 (2.46–12.93)	<.001
Had a positive PCR test for COVID-19 since enrollment visit^c,d^	16 (51.6)	6 (2.3)	46.04 (15.75–134.64)	<.001
**COVID-19 vaccine status**				
Not vaccinated^e^	11 (35.5)	84 (31.7)	Reference	
Fully vaccinated^f^	9 (29.0)	86 (32.5)	0.80 (0.32–2.03)	.56
Partially vaccinated^g^	8 (25.8)	79 (29.8)	0.77 (0.30–2.02)	.51
Missing	3 (9.7)	16 (6.0)	1.43 (0.36–5.71)	.43

a One HCP in the seropositive group was missing a response to this question.

b One HCP in the seropositive group and 1 HCP in the seronegative group were missing a response to this question.

c Follow-up visits were scheduled 2–3 months after the enrollment visit (range, 70–160 d).

d Two HCP in the seronegative group were missing a response to this question.

e Two HCP in the seropositive group and 1 HCP in the seronegative group were missing a response to this question.

f One HCP in the seronegative group received a 1-dose vaccine. All other fully vaccinated HCP received a 2-dose vaccine.

g Vaccine dose information was missing for 1 HCP in the seropositive group and 8 HCP in the seronegative group.

Like the enrollment analysis, having a household member with confirmed or suspected COVID-19 in the time between the enrollment and follow-up visits was associated with seropositivity at follow-up (OR, 9.35; 95% CI, 3.99–21.93) (Table 3). Having another COVID-19 exposure outside work (other than a sick household member) in the time between the enrollment and follow up visits was also a risk factor for being seropositive at follow-up (OR, 4.31; 95% CI, 1.52–12.19) (Table 3). However, unlike at enrollment, having a COVID-19 exposure while at work was not associated with seropositivity at follow-up (OR, 1.46; 95% CI, 0.64–3.36). Like the enrollment analysis, level of contact with COVID-19 patients, use of social distancing or a mask while at work, recent travel, and visits to public locations in the 30 days prior to the follow-up visit were not associated with seropositivity.

SARS-CoV-2 vaccinations were made available to HCP during the beginning of the study follow-up period. By the time of their follow-up visit, 182 HCP (61.5% of those who completed follow-up serology testing and a follow-up survey) had received at least 1 dose of a SARS-CoV-2 vaccine, and 95 (32.1%) had received 2 doses of a 2-dose vaccine. COVID-19 vaccination status was not associated with antibody test results at follow-up (OR, 0.77; 95% CI, 0.32–2.03 for fully vaccinated vs unvaccinated HCP) (Table 3).

## Discussion

In this study, we determined the seroprevalence of SARS-CoV-2 IgG N-antibodies among HCP with no documented history of COVID-19 at 2 time points. We examined IgG anti–N-antibodies to distinguish infection-induced immunity from vaccine response.^5^ At enrollment (September 2020–December 2020), 4.5% of HCP were seropositive, despite having no reported history of a positive SARS-CoV-2 test. At follow-up, 2–3 months later (December 2020–April 2021), 11.0% were seropositive. Among the 34 HCP who had a positive antibody test at follow-up, 9 had also been seropositive at enrollment, 16 reported a positive SARS-CoV-2 PCR test between their enrollment and follow-up visits, and 3 did not provide information about SARS-CoV-2 testing.

Our findings are comparable with community-reported rates of SARS-CoV-2 N-antibody seroprevalence among healthy adults in Missouri in September 2020,^8^ and with previous work from our institution showing that 6.1% of adult patients presenting to St. Louis metropolitan area hospitals were IgG S-antibody (spike protein) seropositive in November of 2020, increasing to 19.03% in January 2021.^9^ In the latter study, S-antibodies were indicative of prior infection because the vaccine was not yet available to the public. Our findings are similar to those from a large multistate study by Self et al, in which 6% of high-risk HCP (range, 0.8%–31.2%) were seropositive for SARS-CoV-2 S-antibodies when tested between April and June 2020.^10^ However, unlike our study, Self et al^10^ tested for antibodies to spike protein and did not exclude HCP with a previous COVID-19 diagnosis, which made up 31% of their cohort. In another study involving blood donors in the Midwest, 15.8% had infection-induced seropositivity to SARS-CoV-2 as of December 2020, which increased to 23.5% by May 2021.^11^ The relatively lower rate of seropositivity among our cohort of HCP at follow-up (11%) is likely due to the initial exclusion of HCP with a known history of COVID-19 from the study cohort; however vaccination efforts, which started in December 2020, may also have contributed.

In our cohort, 61.5% of HCP who completed follow-up serology testing and a follow-up survey reported receiving at least 1 dose of a COVID-19 vaccine prior to their follow-up visit. Although 1 or 2 doses of SARS-CoV-2 vaccine has been reported to prevent both symptomatic and asymptomatic infection in HCP,^12^ vaccination status was not associated with seronegativity in our cohort. This finding may be due to the fact that the SARS-CoV-2 vaccinations only became available to HCP at our facility early during the study follow-up period. Because most HCP had been vaccinated shortly before their follow-up visit, this may have left insufficient time for the full effects of vaccination to be observed in our cohort.

In our cohort, 7 (43.8%) of the 16 HCP who were seropositive at enrollment and who completed a study follow-up visit were seronegative at follow-up. This proportion was higher than that found in a concurrent cohort of SARS-CoV-2 PCR-positive HCP at our institutions, in which 9 (15%) of 62 seropositive HCP experienced seroreversion at 70–180 days follow-up.^13^ This difference may reflect imprecision due to small sample size. In a larger, multistate study, 28.2% of seropositive HCP experienced S-antibody seroreversion ∼60 days after an initial antibody test, and seroreversion was more common (47.9%) among those not reporting prior symptoms.^14^ In our study, the 9 HCP who remained seropositive at follow-up all showed a decline in N-antibody signal over the study period. This observation is consistent with our previous research^13^ and research by others.^15–17^

As has been reported previously in the literature,^18^ having a household contact with SARS-CoV-2 was associated with increased odds of seropositivity among HCP in our cohort at both enrollment and follow-up. At follow-up, having another COVID-19 exposure outside work was also associated with seropositivity. This finding suggests that community exposures are an important risk factor for HCP. In contrast, occupational exposures, including having a direct patient care role and having more frequent contact with COVID-19 patients were not associated with seropositivity in our cohort of HCP.

Our study had several limitations. Because individuals who initially test positive for SARS-CoV-2 antibodies can experience seroreversion in a short time frame, as evidenced both in our cohort and in previous studies,^13,14^ some HCP in our cohort who had a prior SARS-CoV-2 infection may have had antibody levels that had already decreased below the positivity threshold by the time of study enrollment. This factor may have led to underestimation of the baseline prevalence of asymptomatic infections in our cohort. Our study is also limited by the small number of HCP with positive antibody tests at enrollment and at follow-up, which hindered the risk factor analysis, including potential multivariable analysis. Although we attempted to exclude HCP with a known history of COVID-19 at the time of study enrollment, HCP were asked to self-report their SARS-CoV-2 testing history, and we were unable to independently verify this information. Therefore, some HCP who enrolled in the cohort may have had a previous positive SARS-CoV-2 test. Because our study comprised predominately female (64%) and white race (79%) participants, and a large proportion (45%) were physicians, our findings may not be broadly generalizable to healthcare settings with a different demographic composition.

In summary, SARS-Co-V-2 IgG N-antibody testing indicated that ∼5% of HCP who worked with COVID-19 patients during the first wave of the COVID-19 pandemic may have had a previously unknown SARs-CoV-2 infection, similar to rates in the general population. Nonoccupational risk factors, particularly exposure to an infected individual at home, were associated with seropositivity, indicating a need to focus on both occupational and nonoccupational exposures in HCP. Further research is needed to determine whether subclinical SARS-CoV-2 infections result in appreciable immunity to further reinfection or disease or have long-term health implications.

## Supplementary material

For supplementary material accompanying this paper visit http://doi.org/10.1017/ash.2022.375. click here to view supplementary material

## References

[1] WHO coronavirus (COVID-19) dashboard. World Health Organization website. https://covid19.who.int/. Published 2022. Accessed April 7, 2022.

[2] Sah P , Fitzpatrick MC , Zimmer CF , et al. Asymptomatic SARS-CoV-2 infection: a systematic review and meta-analysis. Proc Natl Acad Sci 2021;118:e2109229118. 34376550 10.1073/pnas.2109229118 PMC8403749

[3] Johansson MA , Quandelacy TM , Kada S , et al. SARS-CoV-2 transmission from people without COVID-19 symptoms. JAMA Netw Open 2021;4:e2035057. 33410879 10.1001/jamanetworkopen.2020.35057 PMC7791354

[4] Nalbandian A , Sehgal K , Gupta A , et al. Postacute COVID-19 syndrome. Nat Med 2021;27:601–615. 33753937 10.1038/s41591-021-01283-z PMC8893149

[5] Assis R , Jain A , Nakajima R , et al. Distinct SARS-CoV-2 antibody reactivity patterns elicited by natural infection and mRNA vaccination. NPJ Vaccines 2021;6:132. 34737318 10.1038/s41541-021-00396-3 PMC8568980

[6] Interim guidelines for COVID-19 antibody testing US Centers for Disease Control and Prevention website. https://www.cdc.gov/coronavirus/2019-ncov/lab/resources/antibody-tests-guidelines.html. Published 2022. Accessed July 19, 2022.

[7] Shah VP , Breeher LE , Hainy CM , Swift MD. Evaluation of healthcare personnel exposures to patients with severe acute respiratory coronavirus virus 2 (SARS-CoV-2) associated with personal protective equipment. Infect Control Hosp Epidemiol 2022;43:770–774. 33975656 10.1017/ice.2021.219 PMC8144829

[8] Stout RL , Rigatti SJ. Seroprevalence of SARS-CoV-2 Antibodies in the US adult asymptomatic population as of September 30, 2020. *JAMA Netw Open* 2021;4:e211552. 10.1001/jamanetworkopen.2021.1552 PMC7967079 33724387

[9] Smith BK , Janowski AB , Fremont AC , et al. Progression of SARS-CoV-2 seroprevalence in St. Louis, Missouri, through January 2021. *mSphere* 2021;6:e0045021. 10.1128/mSphere.00450-21 PMC8386375 34346705

[10] Self WH , Tenforde MW , Stubblefield WB , et al. Seroprevalence of SARS-CoV-2 among frontline healthcare personnel in a multistate hospital network—13 academic medical centers, April–June 2020. Morb Mortal Wkly Rep 2020;69:1221–1226. 10.15585/mmwr.mm6935e2 PMC7470460 32881855

[11] Jones JM , Stone M , Sulaeman H , et al. Estimated US infection- and vaccine-induced SARS-CoV-2 seroprevalence based on blood donations, July 2020–May 2021. *JAMA* 2021;326:1400–1409. 10.1001/jama.2021.15161 PMC8414359 34473201

[12] Hall VJ , Foulkes S , Saei A , et al. COVID-19 vaccine coverage in health-care workers in England and effectiveness of BNT162b2 mRNA vaccine against infection (SIREN): a prospective, multicentre, cohort study. Lancet 2021;397:1725–1735. 33901423 10.1016/S0140-6736(21)00790-X PMC8064668

[13] Bosserman RE , Farnsworth CW , O’Neil CA , et al. Antibodies in healthcare personnel following severe acute respiratory syndrome coronavirus virus 2 (SARS-CoV-2) infection. Antimicrob Steward Healthc Epidemiol 2022;2:e93. 36483363 10.1017/ash.2022.231 PMC9726486

[14] Self WH , Tenforde MW , Stubblefield WB , et al. Decline in SARS-CoV-2 antibodies after mild infection among frontline healthcare personnel in a multistate hospital network—12 states, April–August 2020. Morb Mortal Wkly Rep 2020;69:1762–1766. 10.15585/mmwr.mm6947a2 PMC7727600 33237893

[15] Crawford KHD , Dingens AS , Eguia R , et al. Dynamics of neutralizing antibody titers in the months after severe acute respiratory syndrome coronavirus 2 infection. J Infect Dis 2021;223:197–205. 33535236 10.1093/infdis/jiaa618 PMC7543487

[16] Patel MM , Thornburg NJ , Stubblefield WB , et al. Change in antibodies to SARS-CoV-2 over 60 days among healthcare personnel in Nashville, Tennessee. JAMA 2020;324:1781–1782. 32940635 10.1001/jama.2020.18796 PMC7499233

[17] Ibarrondo FJ , Fulcher JA , Goodman-Meza D , et al. Rapid decay of anti–SARS-CoV-2 antibodies in persons with mild COVID-19. N Engl J Med 2020;383:1085–1087. 32706954 10.1056/NEJMc2025179 PMC7397184

[18] Saade EA , Zhang X , Noguez JH , et al. Seroprevalence of severe acute respiratory syndrome coronavirus-2 (SARS-CoV-2) among healthcare providers prior to the vaccine era in an integrated midwestern healthcare system. Antimicrob Steward Healthc Epidemiol 2022;2:e47. 36310795 10.1017/ash.2022.29 PMC9614926

