# Supplementary figures and images for: Seroprevalence of severe acute respiratory coronavirus virus 2 (SARS-CoV-2) antibodies among healthcare personnel in the Midwestern United States, September 2020–April 2021
Source: Antimicrob Steward Healthc Epidemiol. 2023 Aug 4;3(1):e133. doi: 10.1017/ash.2022.375 (PMC10428156; doi:10.1017/ash.2022.375)

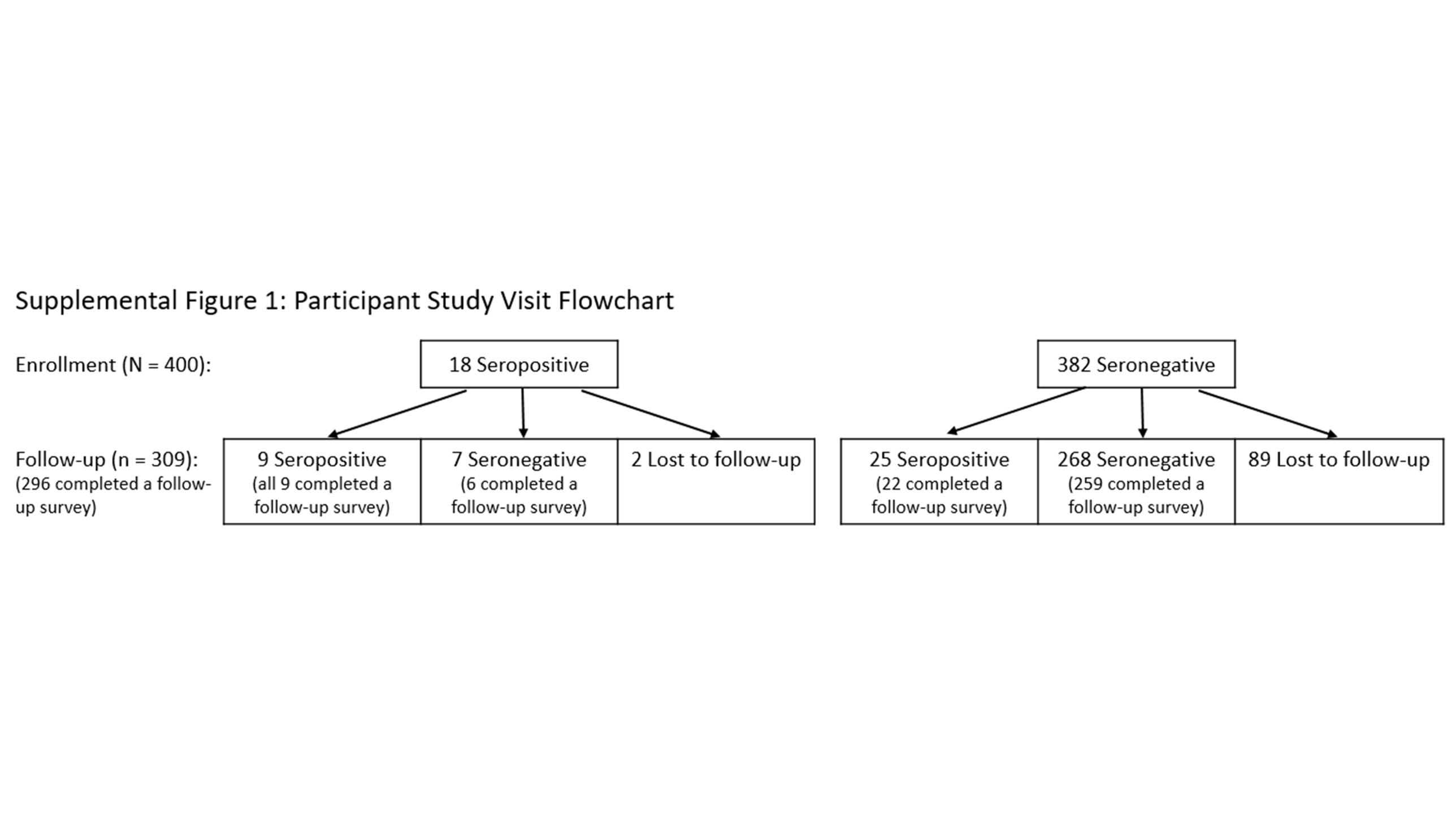

Supplement: Supplementary file 1 [file S2732494X22003758sup001.tif]
